# Metformin may induce ferroptosis by inhibiting autophagy *via* lncRNA H19 in breast cancer

**DOI:** 10.1002/2211-5463.13314

**Published:** 2021-10-27

**Authors:** Jida Chen, Chuan Qin, Yulu Zhou, Yongxia Chen, Misha Mao, Jingjing Yang

**Affiliations:** ^1^ Biomedical Research Center and Key Laboratory of Biotherapy of Zhejiang Province Hangzhou China; ^2^ Department of Pathology Sir Run Run Shaw Hospital Zhejiang University Hangzhou China

**Keywords:** autophagy, breast cancer, ferroptosis, H19, metformin

## Abstract

Autophagy and ferroptosis have been major foci of biomedical research in recent years. Elucidation of their intrinsic molecular relationships is important for cancer prevention and treatment. Metformin can directly inhibit tumorigenesis, although the mechanism responsible for this is not fully understood. Here, we demonstrate that metformin and lncRNA‐H19 can regulate both autophagy and ferroptosis. Autophagy inducers and H19 can reverse the production of lipid reactive oxygen species and the inhibition of autophagy induced by metformin. The present study suggests that metformin may induce ferroptosis by inhibiting autophagy via H19, and this discovery may facilitate the development of novel therapies for the treatment of breast cancer.

## Graphical abstract

The present study demonstrates that metformin can inhibit autophagy and induce ferroptosis, and this can be reversed by H19. Furthermore, the induction of ferroptosis by knockdown of H19 can be reversed by inducing autophagy. These results imply that metformin may induce ferroptosis by inhibiting autophagy through H19, which further highlights the role of H19 in the anticancer effect of metformin.

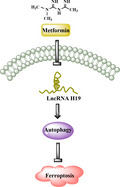

**Abbreviations**
CCK‐8: cell counting kit‐8
GSH: glutathione
ROS: reactive oxygen species
siRNA: small interfering RNA

Despite significant improvements in prevention, diagnosis and treatment, breast cancer is still the most common cancer affecting women’s health and has the highest incidence and the second highest mortality rate of malignant tumors among women [1, 2]. Increasing in‐depth research has provided considerable clinical evidence indicating that metformin has great potential in the treatment of breast cancer [3, 4]. The clinical efficacy, validation and mechanistic exploration of metformin are hotspots in the field of cancer research. Further exploration of the molecular mechanism of metformin anti‐tumor will contribute to its more accurate targeted therapy.

As a lncRNA, the approximately 2.6 kb H19 is a long‐chain non‐coding RNA. It is mainly distributed in the cytoplasm, with low expression in the nucleus. As one of the earliest imprinting genes identified, H19 was expressed according to maternal allele expression and paternal allele imprinting, showing evolutionary conservation in mammals. H19 is involved in regulating the expression or function of other genes through a variety of mechanisms [5]. The H19 expression level is regulated by many factors, including metformin [6]. H19 plays an important role in the regulation of embryonic development and growth and is closely related to the occurrence of various human genetic disorders. Studies have shown that H19 overexpression promotes the tumorigenic properties of breast cancer cells *in vivo* [7]. It is worth noting that H19 is highly expressed in almost all tumor tissues and plays an important role in tumor development [8, 9]. Therefore, H19 may be a potential target for cancer therapy.

Ferroptosis is emerging as a novel form of programmed cell death that is different from apoptosis, autophagy and necrosis [10]. Ferroptosis is an iron‐dependent form of necrotic cell death marked by oxidative damage to phospholipids and a high expression of unsaturated fatty acids in the membrane that leads to the formation of lipid peroxidation, damaging the membrane structure. The main morphological features of ferroptosis are the shrinkage of mitochondria and an increase in mitochondrial membrane thickness, which can be induced by multiple signaling pathways. The major regulatory elements are involved in iron metabolism and oxidative stress and are clinically related to many diseases, including the formation of tumors, neurodegeneration, stroke and ischemia reperfusion injury [11].

Autophagy is a highly conserved degradation of proteins or organelles in eukaryotes. During autophagy, some damaged proteins or organelles are enveloped by the double‐membrane structure of autophagic vesicles and ultimately sent to the lysosome for degradation: the degradation of small molecules such as amino acids can generate energy or supply chemical products for further use. Autophagy is the basis of the degradation and recycling of cell components. In cells, damaged, aged and dead organelles are broken down, rapidly providing energy and raw materials to maintain the normal operation of cells. Autophagy might indeed promote cell death in specific contexts [12], although the underlying mechanisms responsible for this have remained elusive.

In the present study, we found that metformin may induce ferroptosis by inhibiting autophagy mediated by H19, which further highlights the role of H19 in the anticancer effect of metformin, as well as the clinical significance of ferroptosis in cancer therapy, providing a new target for the treatment of cancer with metformin.

## Materials and methods

### Cell culture

MCF7 cells were cultured in RPMI‐1640 medium supplemented with 10% fetal bovine serum and 10 000 U·mL^−1^ penicillin‐streptomycin in a incubator at 37 °C in an atmosphere of 5% CO_2_. The cell culture medium and supplements were purchased from Thermo Fisher Scientific (Waltham, MA, USA).

### Reagents

Sulfasalazine (#HY‐14655) and ferrostatin‐1 (#HY100579) were purchased from MedChemExpress (Monmouth Junction, NJ, USA). Rapamycin (R0395) was purchased from Sigma‐Aldrich (St Louis, MO, USA). Antibodies targeting Beclin1 (3495S; Cell Signaling Technology, Danvers, MA, USA), LC3 (L7543; Sigma‐Aldrich) and GAPDH (sc‐47724; Santa Cruz Biotechnology, Santa Cruz, CA, USA) were also used.

### qRT‐PCR

Total RNA was extracted using TRIzol reagent and cDNA was synthesized with SuperScript II Reverse Transcriptase (Thermo Fisher Scientific). Quantitative real‐time PCR was performed using SYBR GreenER qPCR SuperMix Universal (Thermo Fisher Scientific) and triplicate samples were run on a MX3000P qPCR system (Stratagene, La Jolla, CA, USA) in accordance with the manufacturer’s instructions. The threshold cycle (Ct) values for each gene were normalized to those of GAPDH, and the 2^−ΔΔCt^ method was used for quantitative analysis. The primers used were:

Q‐H19‐F: ACTCAGGAATCGGCTCTGGAA.

Q‐H19‐R: CTGCTGTTCCGATGGTGTCTT.

Q‐GAPDH‐F: TGTGGGCATCAATGGATTTGG.

Q‐GAPDH‐R: ACACCATGTATTCCGGGTCAAT.

### Small interfering RNA (siRNA) plasmids and transfection

For *in vitro* assays, knockdown of H19 was performed by transfection of cells with siRNA duplex oligos using Lipofectamine 3000 (no. 2189668; Invitrogen, Carlsbad, CA, USA) in OPTI‐MEM (no. 2185849; Gibco, Gaithersburg, MD, USA) overnight. The corresponding siRNA sequences were:

siNC: UUCUCCGAACGUGUCACGUdTdT.

siH19#1: CCGUAAUUCACUUAGAAGAdTdT.

siH19#2: CCUUCUAAACGAAGGUUUAdTdT.

### Cell viability assay

Cell viability was evaluated using Cell Counting Kit‐8 (CCK‐8) (GLPBIO, Montclair, USA). Cells were seeded in a 96‐well plate and, after cell treatments, CCK‐8 reagent was added in accordance with the manufacturer’s instructions. Absorbance was measured at 450 nm using a standard instrument.

### Lipid ROS assay

The relative lipid ROS levels in cells were assessed using C11‐BODIPY dye (D3861; Thermo Fisher Scientific). Cells were treated with 5 µm C11‐BODIPY for 30 min, harvested, washed twice with phosphate‐buffered saline and then resuspended in 500 μL of phosphate‐buffered saline. Oxidation of the polyunsaturated butadienyl portion of the dye results in a shift of the fluorescence emission peak from approximately 590 nm to 510 nm.

### Autophagy assay

The autophagic vacuoles were quantified using a cyto‐ID autophagy detection kit (ENZ‐51031; Enzo Diagnostics, Farmingdale, NY, USA) in accordance with the manufacturer’s instructions. The signals of labeled autophagic vacuoles were analyzed using a flow cytometer with an FL1 channel (488 nm excitation, green).

### Glutathione (GSH) assay

The relative GSH concentration in cell lysates was assessed using a total glutathione assay kit (S0052; Beyotime) in accordance with the manufacturer’s instructions. Briefly, glutathione reductase reduces oxidized glutathione to reduced GSH, which reacts with the chromatin substrate 5,5′‐dithiobis(2‐nitrobenzoic acid) to produce yellow 2‐nitro‐5‐thiobenzoic acid and oxidized glutathione. The combination of the two reactions reveals the total glutathione (oxidized glutathione + GSH) and the intensity of 2‐nitro‐5‐thiobenzoic acid (yellow) represents the amount of total glutathione. Thus, the total glutathione content can be calculated by measuring *A*_412_.

### Statistical analysis

Flow cytometry data were analyzed using flowjo, X.10.0.7r2 (https://www.flowjo.com). Statistical analyses were carried out using excel (Microsoft Corp., Redmond, WA, USA) and prism (GraphPad Software Inc., San Diego, CA, USA) to assess differences between experimental groups. Statistical significance was determined by a two‐tailed, unpaired Student’s *t*‐test with a confidence interval of 95%. *P* < 0.05 was considered statistically significant.

## Results

### Metformin can inhibit autophagy via H19

**Fig. 1:**
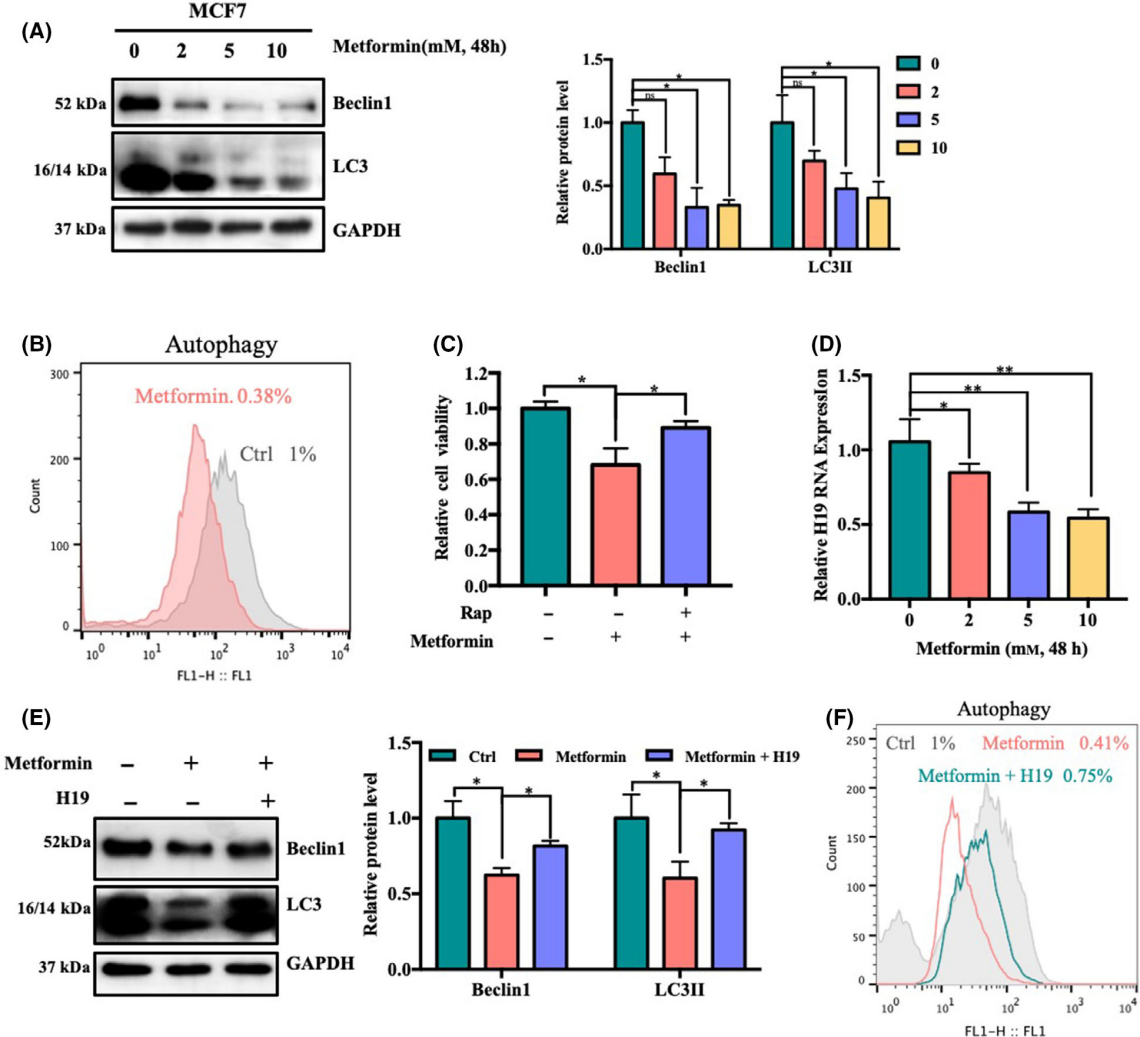
Metformin can inhibit autophagy via H19. (A) MCF7 cells were treated with the indicated concentration (0, 2, 5, or 10 mm) of metformin for 48 h. The protein expression levels of Beclin1 and LC3 were measured via western blotting. Densitometric analysis was performed using imagej (*n* = 3, *P < 0.05, *t*‐test; values represent the mean ± SD). (B) MCF7 cells were treated with 5 mm metformin for 48 h. The level of autophagy was measured by assessing Cyto‐ID green fluorescence. (C) MCF7 cells were treated with metformin (30 mm) in the absence or presence of rapamycin (Rap) (1 μm) for 48 h. Cell viability was assayed (*n* = 3, *P < 0.05, *t*‐test; values represent the mean ± SD). (D) The relative H19 RNA level was measured via qRT‐PCR after MCF7 cells were treated with the indicated concentration (0, 2, 5 or 10 mm) of metformin for 48 h. Values are expressed as the mean ± SD (*n* = 3, *P < 0.05, **P < 0.01, *t*‐test). MCF7 cells were treated with 5 mm metformin for 48 h with or without H19 overexpression. The protein expression levels of Beclin1 and LC3 were measured via western blotting. Densitometric analysis was performed using imagej (*n* = 3, *P < 0.05, t‐test; values represent the mean ± SD). MCF7 cells were treated with 5 mm metformin for 48 h with or without H19 overexpression. The level of autophagy was measured by assessing Cyto‐ID green fluorescence.

MAP1LC3/LC3 (microtubule‐associated protein 1 light chain 3), a mammalian homolog of yeast Atg8, has been used as a marker to monitor autophagy. To determine whether autophagy is involved in the cytotoxicity of metformin, we first examined the Beclin1 protein and the processing of full‐length LC3‐I to LC3‐II, a hallmark of autophagy, in metformin‐treated MCF7 cells. The results showed that metformin inhibited the protein levels of Beclin1 and LC3‐II in a dose‐dependent manner (Fig. 1A). We next examined the change in the autophagic flux. We also observed that metformin blocked autophagic flux in MCF7 cells, confirming that autophagic flux was disrupted in metformin‐treated cells (Fig. 1B). Moreover, the anticancer effect of metformin was inhibited by the autophagy inducer rapamycin (Fig. 1C). These findings demonstrated that metformin acts as an anticancer agent by inhibiting autophagy.

Our laboratory members have published studies showing that metformin can downregulate H19 [6] and also that knockdown H19 can regulate autophagy [13]. Our results have also shown that metformin can inhibit the expression of H19 in MCF7 cell (Fig. 1D). Therefore, we speculate that metformin might regulate autophagy through H19. To test this hypothesis, we overexpressed H19 to detect the effects of metformin on autophagic protein levels and autophagic flow. The results showed that the overexpression of H19 reversed the effects of metformin on the levels of the autophagic proteins LC3 and Beclin1 (Fig. 1E) and autophagic flux (Fig. 1F). Hence, these results suggest that metformin can inhibit autophagy through H19.

### Metformin can induce ferroptosis via H19

**Fig. 2:**
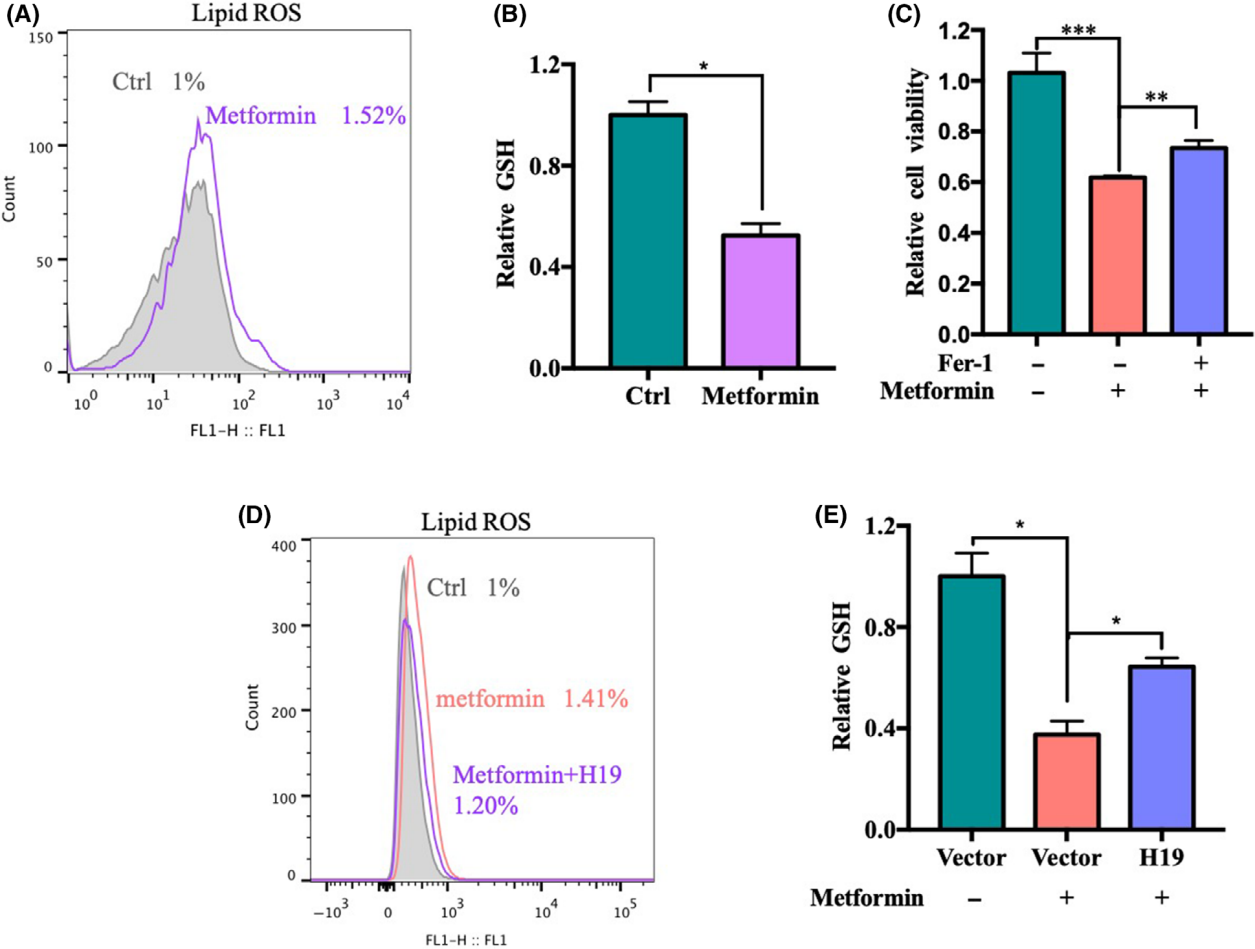
Metformin can induce ferroptosis via H19. (A) MCF7 cells were treated with metformin (5 mm) for 48 h. The relative lipid ROS levels were assayed via C11‐BODIPY fluorescence. (B) MCF7 cells were treated with metformin (5 mm) for 48 h, and the relative levels of GSH were assayed (*n* = 3, *P < 0.05, *t*‐test; values represent the mean ± SD). (C) MCF7 cells were treated with metformin (30 mm) in the absence or presence of ferrostatin‐1 (1 μm) for 48 h. Cell viability was assayed (*n* = 3, **P < 0.01, ***P < 0.001, *t*‐test; values represent the mean ± SD). (D) MCF7 cells were treated with metformin (5 mm) for 48 h with or without H19 overexpression. The relative lipid ROS levels were assayed via C11‐BODIPY fluorescence. (E) MCF7 cells were treated with metformin (5 mm) for 48 h with or without H19 overexpression. The relative levels of GSH were assayed (*n* = 3, *P < 0.05, *t*‐test; values represent the mean ± SD).

Some studies have shown that metformin can trigger the generation of reactive oxygen species (ROS) [14], which is one of the most important signs of ferroptosis. To explore the association between metformin and ferroptosis, we examined the effects of metformin on lipid ROS and GSH, and the results showed that metformin can effectively induce an increase in lipid ROS (Fig. 2A) and inhibit the production of GSH (Fig. 2B). Furthermore, our results showed that the ferroptosis inhibitor ferrostatin‐1 can significantly inhibit the anticancer effect of metformin (Fig. 2C). These findings demonstrated that metformin acts as an anticancer agent by inducing ferroptosis.

Metformin can downregulate H19 (Fig. 1D) [6]. To explore the role of H19 in the regulation of ferroptosis by metformin, we overexpressed H19 to detect the effect of metformin on the levels of lipid ROS (Fig. 2D) and GSH (Fig. 2E). The results showed that the overexpression of H19 could reverse the induction of lipid ROS and the inhibition of GSH mediated by metformin to some extent. These results suggest that metformin can induce an increase in ferroptosis by downregulating H19.

### H19 can regulate autophagy and ferroptosis

**Fig. 3:**
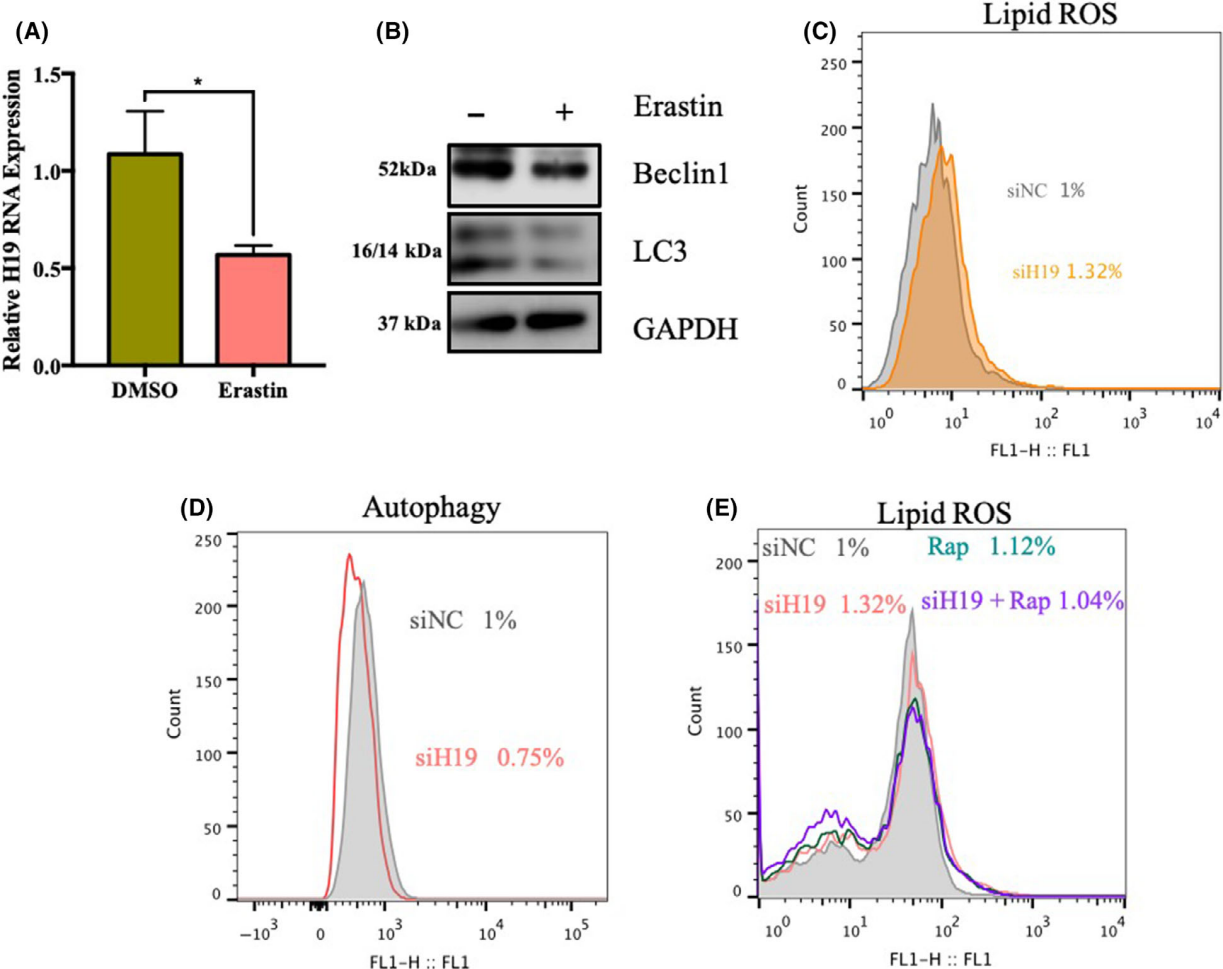
H19 can regulate autophagy and ferroptosis. (A) The relative H19 RNA level was measured via qRT‐PCR after MCF7 cells were treated with erastin (20 μm) for 48 h (*n* = 3, *P < 0.05, *t*‐test; values represent the mean ± SD). (B) MCF7 cells were treated with erastin (20 μm) for 48 h. Beclin1 and LC3 protein expression was measured via western blotting. (C) MCF7 cells were subjected to knockdown of H19. The relative lipid ROS levels were assayed via C11‐BODIPY fluorescence. (D) MCF7 cells were subjected to knockdown of H19. The level of autophagy was measured by assessing Cyto‐ID green fluorescence. (E) MCF7 cells were treated with or without rapamycin after knocking down H19. The relative lipid ROS levels were assayed via C11‐BODIPY fluorescence.

To confirm that H19 is involved in the anticancer effects of metformin, we first investigated whether H19 is involved in regulating ferroptosis. The results show that the ferroptosis inducer erastin can inhibit the expression of H19 (Fig. 3A). Erastin also inhibited the expression of the autophagic proteins Beclin1 and LC3II (Fig. 3B). Because H19 regulates autophagy [4], we therefore speculate that H19 might involve in both the regulation of autophagy and the process of ferroptosis. To further confirm the effects of H19 on ferroptosis and autophagy, we performed a flow analysis using the corresponding kit. The results show that knockdown H19 can induce lipid ROS and inhibit autophagy (Fig. 3C,D). Thus, it is possible that metformin regulates ferroptosis and autophagy via H19.

To further confirm the above conclusion, we downregulated H19 in combination with the use of an autophagy inducer to detect the level of autophagy and the lipid ROS. The results showed that the autophagy inducer rapamycin could reverse the induction of ferroptosis mediated by knocking down H19 (Fig. 3E). These results suggest that downregulation of H19 can inhibit autophagy to induce an increase in ferroptosis.

### Metformin may induce ferroptosis by inhibiting autophagy mediated by H19

**Fig. 4:**
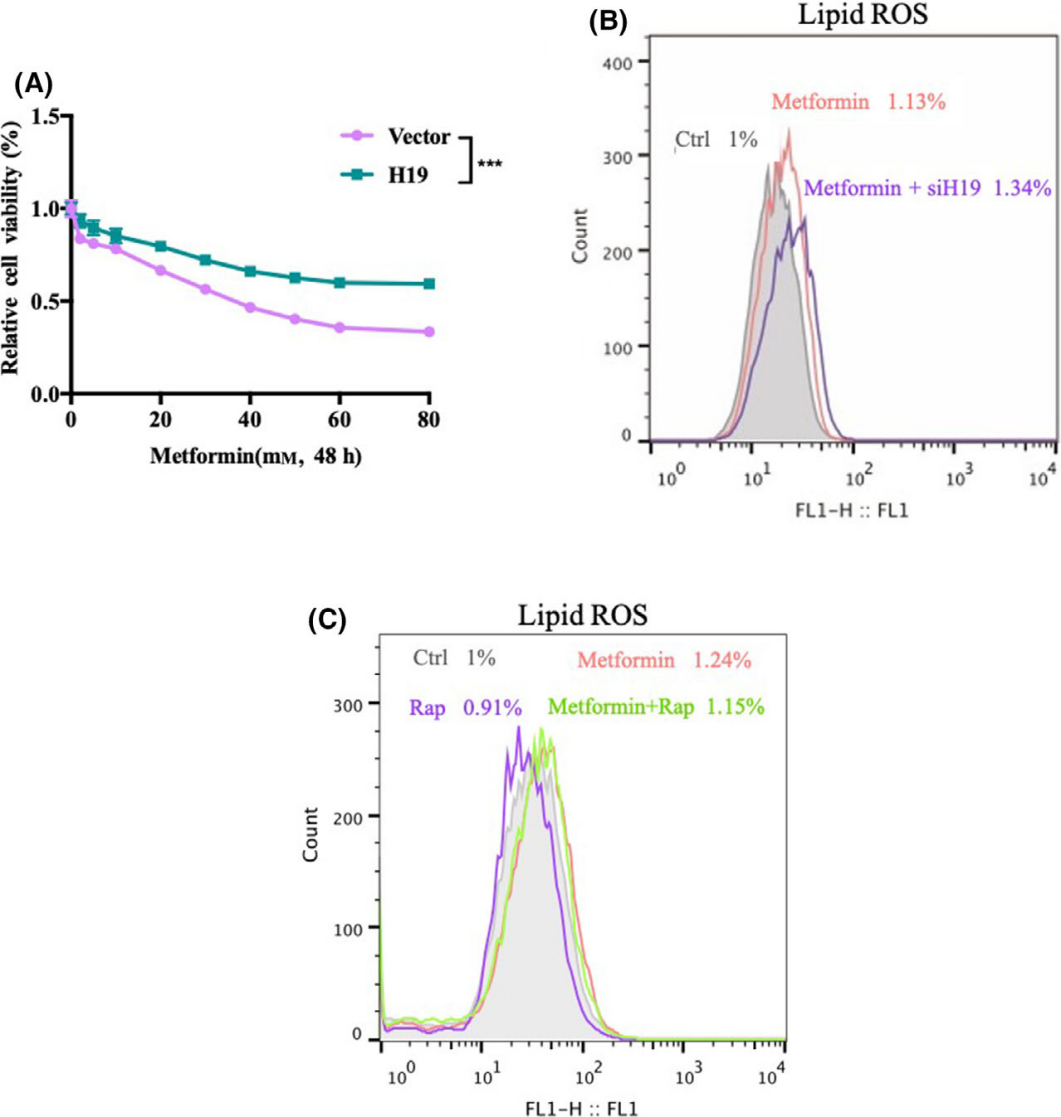
Metformin may induce ferroptosis by inhibiting autophagy mediated by H19. (A) MCF7 cells were treated with metformin (0–80 mm) for 48 h with or without overexpression of H19. Cell viability was assayed (*n* = 3, ***P < 0.001, *t*‐test; values represent the mean ± SD). (B) MCF7 cells were treated with 5 mm metformin for 48 h with or without the knockdown of H19. The relative lipid ROS levels were assayed via C11‐BODIPY fluorescence. (C) MCF7 cells were treated with 5 mm metformin in the absence or presence of rapamycin (100 nm) for 48 h. The relative lipid ROS levels were assayed via C11‐BODIPY fluorescence.

To confirm the involvement of H19 in the antitumor effect of metformin, we examined the effect of H19 overexpression on the toxicity of metformin. The results showed that overexpression of H19 could effectively inhibit the antitumor effect of metformin (Fig. 4A). Interestingly, knockdown H19 contributed to the induction of lipid ROS by metformin (Fig. 4B). To further detect the role of autophagy in metformin‐induced ferroptosis, we treated cells with metformin in combination with the autophagy inducer rapamycin. The results showed that the autophagy inducer rapamycin reduced the metformin‐induced production of lipid ROS (Fig. 4C). All of these results suggest that metformin may induce ferroptosis by inhibiting autophagy via H19.

## Discussion

Metformin, a biguanide commonly used for T2D therapy, can reduce the risk of cancer in diabetic patients to a greater extent than other antidiabetic treatments [15]. Therefore, we aimed to investigate the molecular mechanisms behind the antitumoral action of metformin. Our team has published work showing that metformin can downregulate the expression of H19 [16] and also that H19 can regulate autophagy [13]. Our results further demonstrate that metformin can induce ferroptosis by inhibiting autophagy via H19, which sheds light on the role of H19 in the antitumor effect of metformin and provides new insights into metformin.

Some studies have shown that ferroptosis can be dependent on intracellular iron metabolism, lipid metabolism, and amino acid metabolism [17, 18]. In some cases, autophagy can be mediated through different molecular mechanisms, including degradation of a variety of lipids, proteins and damaged and aged cellular organelles [19]. Although there is some association between ferroptosis and autophagy [20], there is no consensus on the relationship between autophagy and ferroptosis. Many studies have shown that ferroptosis is associated with autophagy activation, although a number of studies have also shown that ferroptosis may be initiated independent of autophagy activation [21]. The relationship between ferroptosis and autophagy remains an open question. In the present study, we have shown that ferroptosis induced by metformin is independent of autophagy, although the specific mechanism by which metformin induces ferroptosis through H19 remains to be further studied. Exploring the specific role of H19 in regulating autophagy and ferroptosis will help to further explain the anticancer mechanism of metformin.

## Conclusions

Our study sheds new light on the idea that metformin induces ferroptosis by inhibiting autophagy through H19, further elucidates the relationship between autophagy and ferroptosis, and identifies a newly discovered role of H19 in regulating ferroptosis. Given that H19 is stable in human plasma and tissues and exhibits good repeatability, it offers great potential as a tumor marker and therapeutic target. Our study provides a theoretical basis for the potential prediction of therapeutic targets of H19 and understanding role of ferroptosis with respect to the antitumor effects of metformin.

## Conflict of interests

The authors declare that they have no conflicts of interest.

## Author contributions

JDC, CQ and JJY designed the study. JJY and MSM performed the western blotting. JJY and YXC performed the cell viability assays. JJY, MSM and YLZ performed the western blotting assay. YXC contributed to technical support. JJY and CQ performed the flow cytometry assay. JJY and JDC analyzed the data and wrote the manuscript. JDC, YXC and JJY supervised the study. All authors read and approved the final manuscript submitted for publication.

## Data Availability

The datasets used and/or analyzed during the present study are available from the corresponding author upon reasonable request.
